# Cold aortic flush after ventricular fibrillation cardiac arrest reduces inflammatory reaction but not neuronal loss in the pig cerebral cortex

**DOI:** 10.1038/s41598-025-95611-9

**Published:** 2025-04-04

**Authors:** Lisa Barones, Wolfgang Weihs, Alexandra Schratter, Andreas Janata, Petra Kodajova, Helga Bergmeister, Lukas Kenner, Michael Holzer, Wilhelm Behringer, Sandra Högler

**Affiliations:** 1 Laboratory Animal Pathology, Department of Biological Sciences and Pathobiology, University of Veterinary Medicine Vienna, Vienna, Austria; 2 Department of Emergency Medicine, Medical University of Vienna, Vienna, Austria; 3 Department of Cardiology, Klinik Floridsdorf, Vienna, Austria; 4 Center for Biomedical Research and Translational Surgery and Ludwig Boltzmann Institute for Cardiovascular Research, Medical University Vienna, Vienna, Austria; 5 Department of Pathology, Department for Experimental and Laboratory Animal Pathology, Medical University of Vienna, Vienna, Austria

**Keywords:** EPR, ECPR, Resuscitation, Neuronal death, Glial response, QuPath, Neuroscience, Medical research

## Abstract

This study aims to retrospectively compare two resuscitation methods *(*extracorporeal cardiopulmonary resuscitation (ECPR) vs. emergency preservation and resuscitation (EPR)) by pathohistologically assessing pig brains in a ventricular fibrillation cardiac arrest (VFCA) model. In prospective studies from 2004 to 2006, swine underwent VFCA for 13 (*n* = 6), 15 (*n* = 14) or 17 (*n* = 6) minutes with ECPR (ECPR13, ECPR15 and ECPR17). Another 15 min VFCA group (*n* = 8) was resuscitated with EPR and chest compressions (EPR15 + CC). Brains of animals surviving for nine days (ECPR13 *n* = 4, ECPR15 *n* = 2, ECPR17 *n* = 1, EPR15 + CC *n* = 7) were harvested. Eight different brain regions were analyzed with the image analysis software QuPath using HE-staining, GFAP- and Iba1-immunohistochemistry. Only ECPR13 and EPR15 + CC animals were included in statistical analysis, due to low survival rates in the other groups. All VFCA samples showed significantly fewer viable neurons compared to shams, but no significant differences between ECPR13 and EPR15 + CC animals were observed. ECPR13 animals showed significantly more glial activation in all cerebral cortex regions compared to shams and in occipital, temporal and parietal cortex compared to EPR15 + CC. In conclusion, EPR + CC resulted in a significantly reduced inflammatory reaction in cerebral cortex compared to ECPR but did not influence the extent of neuronal death after VFCA.

**Supplementary Information:**

The online version contains supplementary material available at 10.1038/s41598-025-95611-9.

## Introduction

Sudden cardiac death is a major health problem. In Europe, the incidence of out of hospital cardiac arrest ranges from 49/100.000 to 56/100.000, with a survival rate of only 8–10% ^1^.

Various new resuscitation methods have been explored in cardiac arrest (CA) animal models with the goal to improve outcome after cardiac arrest in humans. Extracorporeal cardiopulmonary resuscitation (ECPR) was first explored in animal studies^2,3^, and showed promising results in recent human studies^4^. A more advanced approach with rapid induction of cerebral hypothermia by cold aortic flush before resuscitation (Emergency Preservation and Resuscitation, EPR) is another promising method in animal models to improve outcome after exsanguination CA^5,6^, as well as after normovolemic CA^7,8^.

The most common outcome parameters in CA animal models are neurologic and behavioral score systems as well as assessment of histologic damage in brain regions, primarily the hippocampus^9–12^. Although it has been shown that multiple brain regions are selectively vulnerable cross-species to ischemic damage and reperfusion injury after CA^13,14^, only few publications present more detailed histopathological evaluation using quantitative image analysis software (e.g., QuPath, ImageJ or Fiji)^15,16^.

In two previous swine cardiac arrest studies, we found that good functional outcome can be achieved with ECPR after a maximum cardiac arrest no-flow time of 13 min^10^, and with EPR after a cardiac arrest no-flow time of 15 min^9^. The aim of this retrospective study was to investigate the effects of resuscitation with ECPR and EPR on neuronal necrosis and inflammatory reaction in various selectively vulnerable brain regions of brains harvested from the two previous studies^9,10^ by using a quantitative image analysis software. Instead of using semiquantitative score systems, such as the histological damage score (HDS)^17^, quantitative neuron counting in hematoxylin and eosin (HE) staining was chosen to analyze the extent of neuronal necrosis. The degree of inflammatory reaction was evaluated by quantitative pixel counting in immunohistochemistry with primary antibodies against ionized calcium-binding adapter molecule 1 (Iba1), and glial fibrillary acidic protein (GFAP), indicating microglia and astrocytes respectively. Since the two resuscitation methods enabled good functional outcome after different no flow durations, we hypothesized that the extent of neuronal necrosis and inflammatory reaction in various brain regions depends on the resuscitation method.

## Methods

The tissue investigated in this study was stored from studies performed in 2004 to 2006 ^9,10^ on female swine (large white breed, 28–37 kg) supplied by the pig farm (Medau, Austria) of the University of Veterinary Medicine Vienna. These studies were approved by the Institutional Ethics Committee for Laboratory Animal Experiments of the Medical University of Vienna and the Federal Ministry of Education, Science and Research of Austria GZ 66.009/42-Pr/4/2002. All methods in this study were performed in accordance with the relevant guidelines and regulations. The study is reported in accordance with the ARRIVE guidelines, with the exception of animal randomization, due to the distinct technical setup required for each resuscitation method. Animals were delivered two weeks prior to the start of the experiment and were housed in groups with a 12 h/12 h light/dark cycle. Water and conventional swine food were provided ad libitum. Eight animals from a training surgery at the Department of Biomedical Research, Medical University of Vienna lasting three to four hours without any cardiac arrest time, were used as sham animals.

### Animal preparation

CA swine were anesthetized with a premedication of midazolam (1.25 mg/kg), acepromazine (1.75 mg/kg) and atropine (0.5 mg). Analgesic and antibiotic prophylaxis were covered with piritramide (15 mg) and enrofloxacin (5 mg/kg). For intubation the anesthesia was deepened with propofol (40–80 mg), swine were mechanically ventilated thereafter. Physiological saline was administered, and the maintenance of the anesthesia was achieved with propofol (20 mg/kg/h) and boluses of piritramide (30 mg) intravenously via a peripheral venous ear cannula. Animals were equipped with arterial and pulmonary artery access to measure mean arterial pressure (MAP), central venous pressure (CVP), pulmonary artery pressure (PAP) and pulmonary capillary wedge pressure (PCWP) as well as pulmonary artery temperature (T_p.a._). Baseline brain temperature was measured with a brain temperature probe inserted into the frontal lobe. An arterial and venous bypass cannula were inserted into the right femoral vein and artery, respectively. In cold aortic flush groups, a balloon catheter was additionally inserted into the left femoral artery and advanced to 47.5 cm from the insertion side^9,10^.

Sham animals were anesthetized with midazolam (1.25 mg/kg), acepromazine (1.75 mg/kg) and atropine (0.5 mg). For intubation the anesthesia was deepened with propofol (40–80 mg) and maintained with sevoflurane and fentanyl (20 µg/kg/h). Muscle relaxation was achieved with rocuronium (1,5 mg/kg/h), and hydration was maintained with ringer solution (600 ml/h). Noradrenaline (1–4 µg/kg/h) was given if required. The pigs underwent a training surgery involving endoscopic procedures within the abdominal cavity for 3–4 h. After the training they were euthanized by a propofol bolus followed by a potassium overdose. The brain was taken and fixed in formalin immediately. These animals were neither subjected to a cardiac arrest nor to any resuscitation procedures involving ECPR or EPR.

### Cardiac arrest

An external electrical impulse of 90 J triggered the VFCA, which was confirmed by a drop in blood pressure and ventricular fibrillations in the electrocardiogram (ECG). The mechanical ventilation was disconnected and animals stayed in VFCA for 13, 15–17 min, respectively^9,10^.

### Resuscitation

After 13, 15, or 17 min of no-flow time, advanced life support with chest compressions at a compression rate of 90 per minute was started and epinephrine, and vasopressin in combination with heparin were administered in all animals. For ECPR, cardiopulmonary bypass (CPB) was initiated after 20 min of cardiopulmonary resuscitation (CPR) with a flow rate of 70 ml/kg/min and 100% oxygen. The aimed PaO_2_ was 80–120 mmHg, PaCO_2_ 30–40 mmHg, and aimed MAP was 80 mmHg. Cooling to the aimed core temperature (34.5 °C) was initiated with CPB. For EPR, a flush of 150 ml/kg ice cold (4 °C) saline with a flow rate of 1.2 l/min was given via roller pump into the thoracic aorta through an aortic balloon catheter, and 50 ml/kg saline (4 °C) with a flow rate of 30 ml/kg/min was given into the femoral artery (flush) through the bypass cannula before starting CPR. For edema prevention, the right heart was vented during the flush, and the first 300 ml blood were collected in citrate bags. During CPB this blood was reinfused. The flush took around five minutes, contained epinephrine, vasopressin, and heparin and induced a deep cerebral hypothermia (13.3 ± 6.0 °C). During CPR the brain temperature increased until it reached the aimed core target temperature of 34.5 °C within the first minutes of CPB. In all animals defibrillation attempts, with biphasic countershocks of 150 J, were started after at least four minutes CPB. Defibrillation attempts were continued for up to an hour unless ROSC could be achieved and the flow rate of the CPB could be turned down to 15 ml/kg/min. After ROSC, CPB was continued for about 15 min, swine were weaned from bypass and transferred to an intensive care unit afterwards. The average times from the CA to ROSC, the average full-flow CPB times and the average low-flow CPB times of each group are summarized in Table 1 (Table 1)^9,10^.

**Table 1:** Average CA to ROSC, full-flow CPB, and low-flow CPB time.

Group	Average time CA to ROSC	Average full-flow (70 ml/kg/min) CPB time	Average low-flow (15 ml/kg/min) CPB time
13 min CA and ECPR (ECPR13)	34.5 ± 5.2 min	5.25 ± 1.89 min	18.75 ± 6.95 min
15 min CA and ECPR (ECPR15)	40.0 ± 0 min	5.0 ± 1.0 min	16.0 ± 2.0 min
17 min CA and ECPR (ECPR17)	51.0 min	6.0 min	12.0 min
15 min CA and EPR with chest compressions (EPR15 + CC)	42.67 ± 5.32 min	4.17 ± 0.41 min	17.00 ± 3.58 min

### Intensive care

All animals were rewarmed after 16 h of targeted temperature management (TTM, 34.5 °C) and stayed in the intensive care unit for about 20 h, after which they were extubated and returned to the animal house. If the respiratory and neurologic situation was not adequate to wean from ventilation and sedation, the swine was euthanized on day four of survival. Otherwise, the animals were euthanized on day nine^9,10^.

### Group assignment

**Fig. 1:**
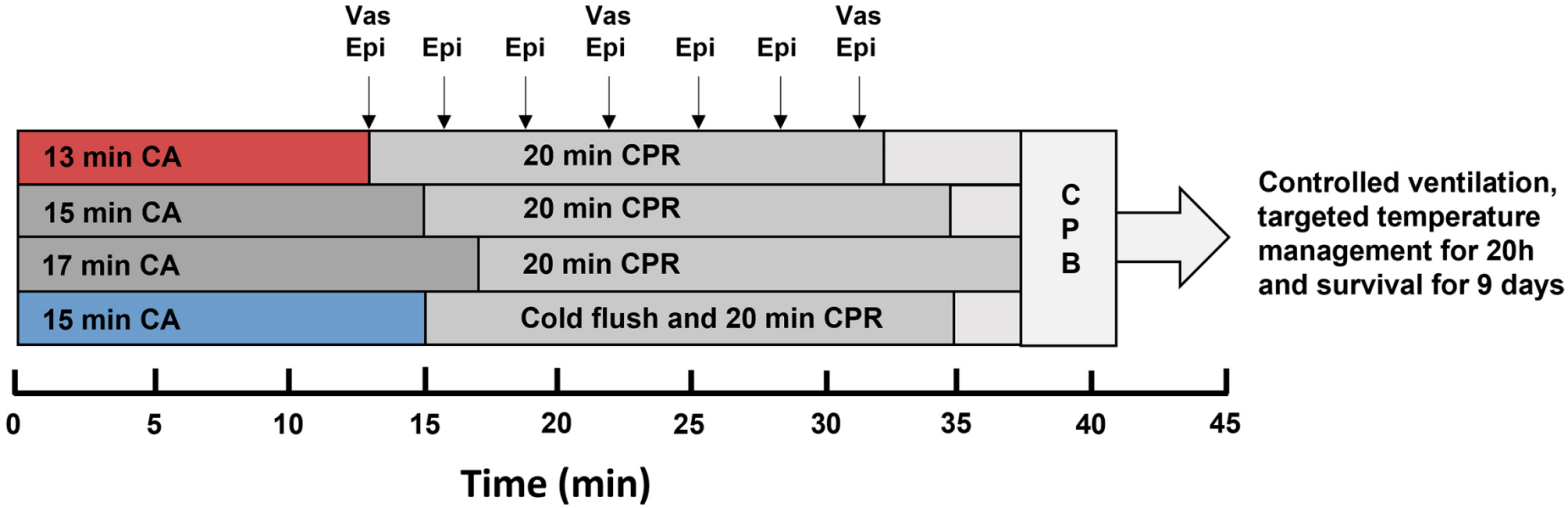
Group assignment. ECPR groups with 13 (in red), 15, or 17 min of cardiac arrest (CA), EPR + CC group with 15 min of CA (in blue) receiving 150 ml/kg ice cold saline into the thoracic aorta and 50 ml/kg into the femoral artery. All groups were treated with 20 min of cardio-pulmonary resuscitation (CPR) accompanied by epinephrin (Epi) and vasopressin (Vas) injections. Thereafter, cardio-pulmonary bypass (CPB) was activated, countershocks were applied and, following restoration of spontaneous circulation, CPB was discontinued. Animals were kept at a target temperature of 34.5 °C for 16 h and survived for nine days.

In total six pigs were resuscitated with ECPR in the 13 min group (ECPR13), 14 pigs in the 15 min group (ECPR15), and six pigs in the 17 min group (ECPR17). Eight swine were included into the 15 min EPR group (EPR15 + CC) receiving ice-cold flush and chest compressions. Only animals, which had ROSC and survived till nine days post resuscitation, were included into the present study and compared to sham animals with three to four hours anesthesia (sham) (Fig. 1).

### Tissue preparation

On the day of final examination, animals were euthanized using the following protocol: The animals were re-anesthetized and mechanically ventilated as described in animal preparation (midazolam and piritramide, anesthesia with propofol). The aorta was cannulated and clamped distal of the cannulation during a thoracotomy, while the right atrium was opened. Brain and heart were perfused with four liters of saline, followed by one liter of 3,7% buffered formaldehyde solution (pH 7.4), which led to the death of the animals. Brains were postfixed, cut in coronary sections and embedded in paraffin^9,10^.

After collecting and checking all paraffin embedded samples, formaldehyde-fixed tissue was checked for samples, which were missing in paraffin. Formaldehyde-fixed material (21 of 98) was used to complete the sample set and perform the following retrospective study. Coronary sections of fixed brain tissues were cut at the level of striatum, thalamus, hippocampus, and cerebellum. Material was embedded in paraffin and cut into sections of 2 μm thickness, which were stained with hematoxylin and eosin (HE), and immunohistochemistry (IHC) was performed with an autostainer (Autostainer Lab Vision 360, Thermo Fisher Scientific, Waltham, USA). Microglia and astrocytes were detected using primary antibodies against ionized calcium-binding adapter molecule 1 (Iba1) and glial fibrillary acidic protein (GFAP), respectively. Details regarding antibodies, pretreatment and dilution are presented in the supplemental Table 1. (suppl. data, Table 1).

Since a retrospective study was performed, material of a few regions in some animals was missing, and could not be examined.

### QuPath evaluation

In total 331 slides in HE-staining and two immunohistochemical stainings, containing eight different brain regions, prior estimated as selectively vulnerable to ischemia, namely frontal, parietal, temporal and occipital cortices, putamen, caudate nucleus, hippocampus, and cerebellar cortex were examined for this retrospective analysis. Furthermore, medullae of the cerebral and cerebellar cortical regions were examined by immunohistochemistry and analyzed separately.

All slides were scanned as whole slide scans with a whole slide imaging scanner (Panoramic SCAN II SC150-213005, 3DHISTECH KFT, Budapest, Hungary)^18^ with a 20x objective, and analyzed with the software platform for bioimage analysis, QuPath, Version 0.2.3 for Windows (QuPath-Quantitative Pathology & Bioimage Analysis, freeware, University of Edinburgh, UK)^19^.

Each region of interest (ROI) in HE-staining was given five randomly set areas of 350 × 550 μm, wherein the number of viable neurons was determined. Only neurons with clearly definable nucleus were counted. In the cerebellar cortex, counting of neurons was limited to the Purkinje cells. In the cerebral cortex, three areas were randomly set between layers I to III and two areas between layers IV to VI.

In immunohistochemical stainings, putamen, caudate nucleus, and the CA1 and CA2 regions of the hippocampus were defined as ROI, respectively. In cortical regions (frontal, parietal, temporal, and occipital) one gyrus, and in the cerebellum one folium was defined, divided into medulla (white matter) and cortex (gray matter), and analyzed separately. ROIs were evaluated by positive pixel counting, whereby the downsample factor and gaussian sigma, as well as thresholds for hematoxylin and 3,3’-diaminobenzidine (DAB) staining, were defined for each ROI individually. Results were expressed as percentage of positive pixels (brown staining) out of all stained pixels (counterstain) within the ROI. In this study one hemisphere per region was evaluated, due to no significant differences between hemispheres in previous study results^17^. Representative images of the figures were taken with QuPath.

### Statistical analysis

For HE data the average value out of the five ROI per region was calculated. All outliers were excluded from calculations, and data were checked for normal distribution with the Shapiro-Wilk-Test. If data were normally distributed, an ANOVA (Analysis of Variance) test was performed for determination of significant differences between groups, otherwise the Kruskal-Wallis-Test was used. Tukey’s range test or Dunn´s test was used for correction of multiple comparisons. Calculations were carried out with GraphPad Prism version 9.3.1 (471) for Windows (GraphPad Software, San Diego, USA). Normally distributed data are presented as mean and standard deviation. Not normally distributed data are presented as median and interquartile range.

## Results

### Survival

**Fig. 2:**
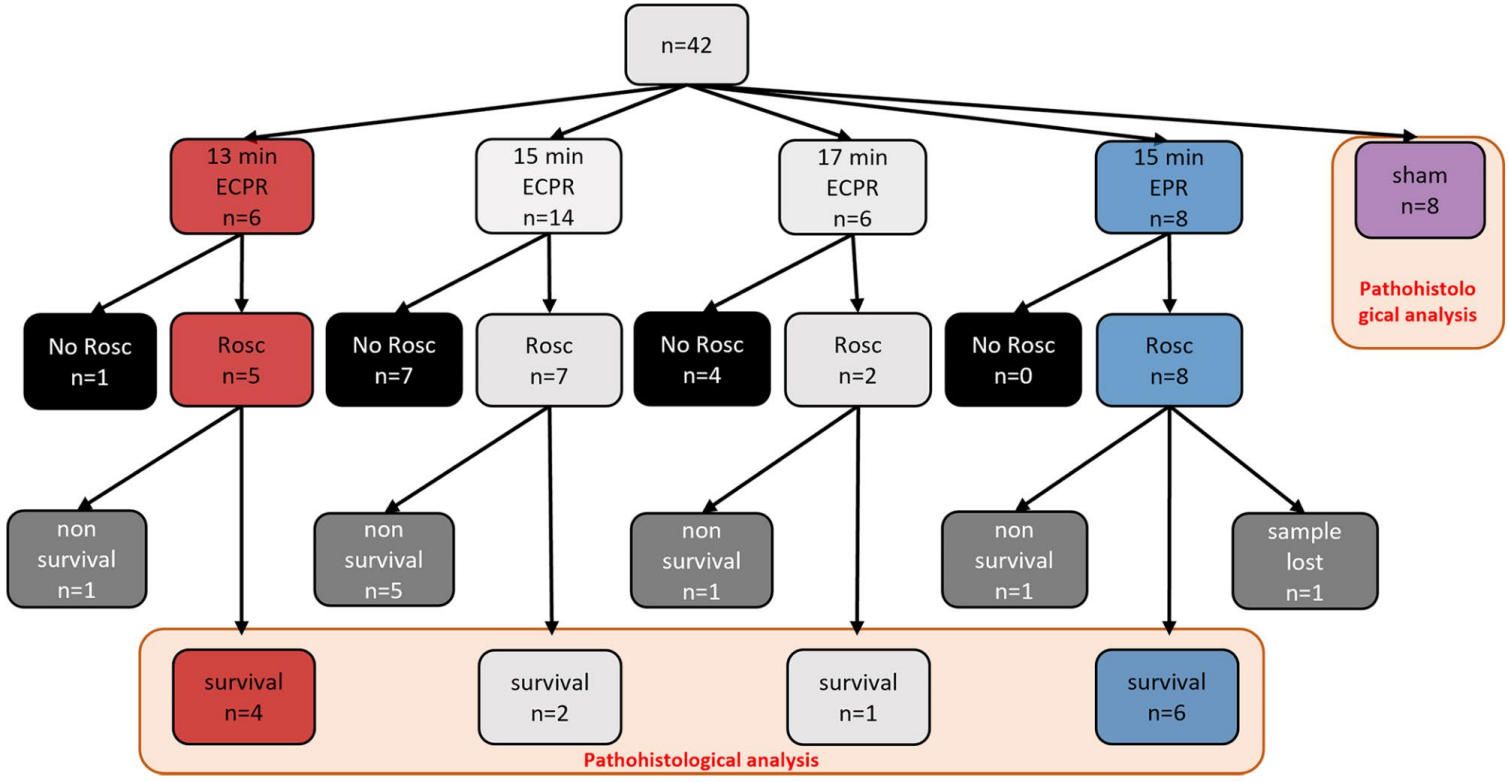
Overview of ROSC achievement and survival of all animals examined. Animals subjected to 13 min of VFCA, resuscitated with ECPR (in red) and animals subjected to 15 min of VFCA, resuscitated with EPR (in blue) and surviving to the endpoint after nine days of survival were compared to sham animals (in purple). Numbers of animals used for pathohistological analysis encircled in light orange.

In the ECPR13 group four animals out of six survived till day nine. In the ECPR15 group only two out of 14, and in the ECPR17 group only one pig out of six survived till day nine post resuscitation^9,10^. In comparison, seven pigs survived the whole nine days in the EPR15 + CC group (Fig. 2)^9^.

All surviving animals of the ECPR13 (*n* = 4) and the EPR15 + CC (*n* = 6) group were histologically and statistically evaluated. Due to the low survival rate of the ECPR15 and ECPR17 group, we excluded these animals from the statistical analysis, but they were still evaluated, and results are presented in the supplementary data. Also, one animal of the EPR + CC group had to be excluded because of missing brain material.

### Histological examination

The results of HE-staining and immunohistochemistry are presented jointly for the respective regions.

### Cerebral cortex

**Fig. 3:**
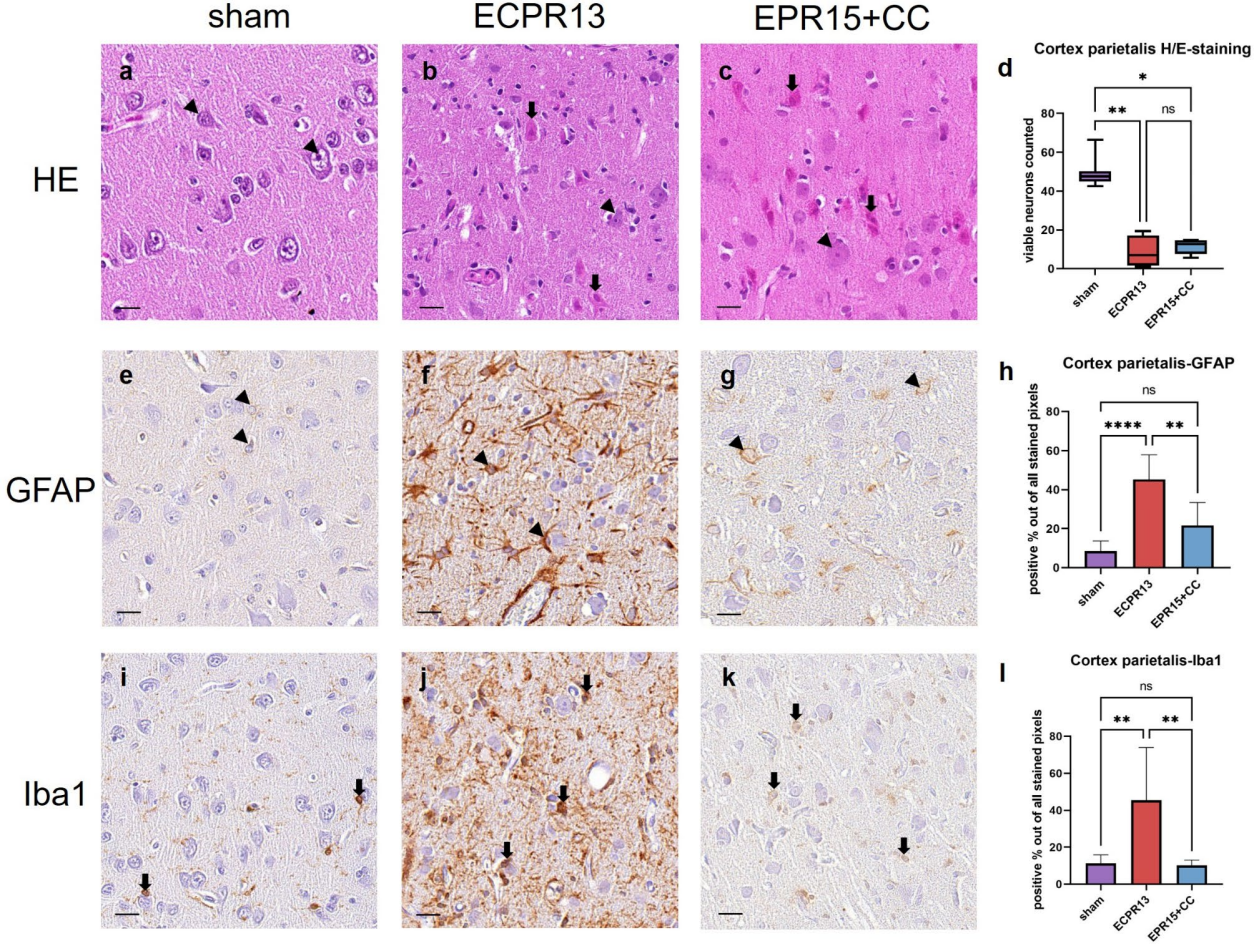
Representative images of the parietal cortex in HE-staining, Iba1- and GFAP-immunohistochemistry. (**a**-**d**) HE-staining, parietal cortex: **a** physiological appearance of layer III in a sham animal with large pyramidal neurons (arrowheads); (**b**) multiple necrotic neurons (thick arrows) and scattered viable neurons (arrowheads) in an ECPR13 animal; (**c**) EPR15 + CC animal, multiple necrotic neurons (thick arrows) and few viable neurons (arrowheads); (**d**) the number of viable neurons in parietal cortex of ischemic groups was significantly lower compared to sham animals in HE-stained samples (**p = 0.0067; *p = 0.0115). (**e-h**) GFAP-immunohistochemistry, parietal cortex: (**e**) astrocytes showed very little cytoplasmic staining (arrowheads) in a sham animal; (**f**) highly activated astrocytes (arrowheads) were present in an ECPR13 animal; (**g**) signal in lesser extent and intensity in an EPR15 + CC pig (arrowheads); (**h**) GFAP expression was significantly higher in ECPR13 pigs compared to sham pigs (****p < 0.0001) and EPR15 + CC animals (**p = 0.0053). (**i-l**) Iba1-immunohistochemistry, parietal cortex: (**i**) physiologic Iba1 expression in microglial cells (thick arrows) in a sham animal; (**j**) highly increased Iba1 expression (thick arrows) in an ECPR13 animal; (**k**) mild Iba1-expression (thick arrows) in an animal of the EPR15 + CC group; (**l**) significantly higher Iba1-expression was present in parietal cortex of ECPR13 pigs compared to sham animals (**p = 0.0046) and EPR15 + CC pigs (p=**0.0098). ns = no significant differences, bars = 20 μm.

Sham animals showed physiological neurons with slightly basophil cytoplasm, a round, clear definable nucleus and one central nucleolus in HE-staining. All six layers of the cerebral cortex (I-VI) could be distinguished. Two of four ECPR13 pigs showed marked neuronal necrosis, edema, and cavitation, while others had primarily moderate necrosis, and edema with mild or no cavitation evident. EPR15 + CC pigs also showed primarily mild to moderate neuronal necrosis and edema, but hardly any cavitation except for the frontal cortex, which showed more severe lesions. Representative images of the parietal cortex are shown in Fig. 3a-c. The number of viable neurons in all cortical regions was significantly higher (parietal cortex, Kruskal-Wallis, *p* = 0.0067 and *p* = 0.0115; temporal cortex, Kruskal-Wallis, *p* = 0.0086 and 0.0096; frontal cortex and occipital cortex, ANOVA, *p* < 0.0001) in sham animals (*n* = 8) compared to animals subjected to VFCA (ECPR13 *n* = 4 and EPR15 + CC *n* = 6), regardless of the resuscitation method (Fig. 3d; suppl. data, Fig. S1a-d).

In GFAP staining astrocytes in the cerebral cortex of sham animals appeared as small, round, and slightly counterstained nuclei, with very little cytoplasm and fine processes. The majority of the ECPR13 animals showed highly activated astrocytes, with a high amount of dark brown cytoplasm, and abundant positively stained dots appeared in the neuropil, due to cell processes cut perpendicularly. In EPR15 + CC pigs, astrocytes exhibited thin and light brown processes and very little cytoplasm. Representative images of the parietal cortex are shown in Fig. 3e-g. GFAP-immunohistochemistry showed significantly more and higher activation of astrocytes (parietal cortex and occipital cortex, ANOVA, *p* < 0.0001; temporal cortex, ANOVA, *p* = 0.0033; frontal cortex, ANOVA, *p* = 0.0002) in all cerebral cortex regions of ECPR13 animals (*n* = 4) compared to sham animals (*n* = 8). In contrast, in the parietal and temporal cortex of EPR15 + CC pigs (*n* = 6) GFAP staining was not significantly (*p* ≥ 0.05) increased compared to sham animals (*n* = 8). Furthermore, EPR15 + CC animals (*n* = 6) showed significantly less staining (parietal cortex, ANOVA, *p* = 0.0053; temporal cortex, ANOVA, *p* = 0.0240; occipital cortex, ANOVA, *p* = 0.0440) compared to ECPR13 animals (*n* = 4) in all cortex regions, except for the frontal cortex (Fig. 3h; suppl. data, Fig. S2, a-d).

Resting microglia cells of sham animals showed positive cytoplasmic signal with thin processes in all cerebral cortex regions. ECPR13 pigs presented with reactive microglia, characterized by increased dark brown cytoplasm, thicker but fewer processes, and rod-shaped nuclei. The neuropil appeared dark brown, due to cut cell processes. Animals in the ECPR13 group showed high interindividual variability in all cortex regions, which led to high standard deviations in the quantitative software analysis. Nevertheless, significantly more, and higher activation of microglia (parietal cortex, ANOVA, *p* = 0.0046; frontal cortex, ANOVA, *p* = 0.0011; occipital cortex, Kruskal-Wallis, *p* = 0.0140) was present in all cortical regions but the temporal cortex in the ECPR13 animals (*n* = 4) compared to sham (*n* = 8). In contrast, no differences were detectable between EPR15 + CC and sham animals in any cortex region (suppl. data, Fig. S2, i-l). Furthermore, EPR15 + CC pigs (*n* = 4) showed significantly fewer positive pixels in the parietal cortex (ANOVA, *p* = 0.0098) compared to ECPR13 pigs (*n* = 4) (Fig. 3i-l).

### Cerebral medulla

The white matter does not contain neurons; therefore, an evaluation of the cerebral medulla was performed only in immunohistochemistry. Resting and activated astrocytes and microglia cells in the white matter of all examined neocortical regions showed similar appearance and shape as described in the cortices. In GFAP staining, no significant differences (*p* ≥ 0.05) among groups could be found, except for significantly fewer positive pixels (ANOVA, *p* = 0.0159) in EPR15 + CC pigs (*n* = 6) compared to ECPR13 pigs (*n* = 4) in the occipital medulla (suppl. data. Fig. S2, e-h).

Iba1 staining of frontal, temporal, and occipital white matter showed significantly more signal (frontal cortex, ANOVA, *p* < 0.0001; temporal cortex, ANOVA, *p* = 0.0086; occipital cortex, ANOVA, *p* = 0.0001) in ECPR13 pigs (*n* = 4) compared to sham animals (*n* = 8). No significant differences (*p* ≥ 0.05) were detectable between sham and EPR15 + CC animals in parietal, temporal and occipital cortex. The frontal medulla revealed significant differences (ANOVA, *p* < 0.0001, *p* = 0.0189 and *p* = 0.0179) between all groups examined (EPR15 + CC *n* = 6, sham *n* = 8), with the highest positive signal in ECPR13 animals (*n* = 4). The parietal medulla showed no significant (*p* ≥ 0.05) differences among groups (suppl. data, Fig. S2, m-p).

### Caudate nucleus

**Fig. 4:**
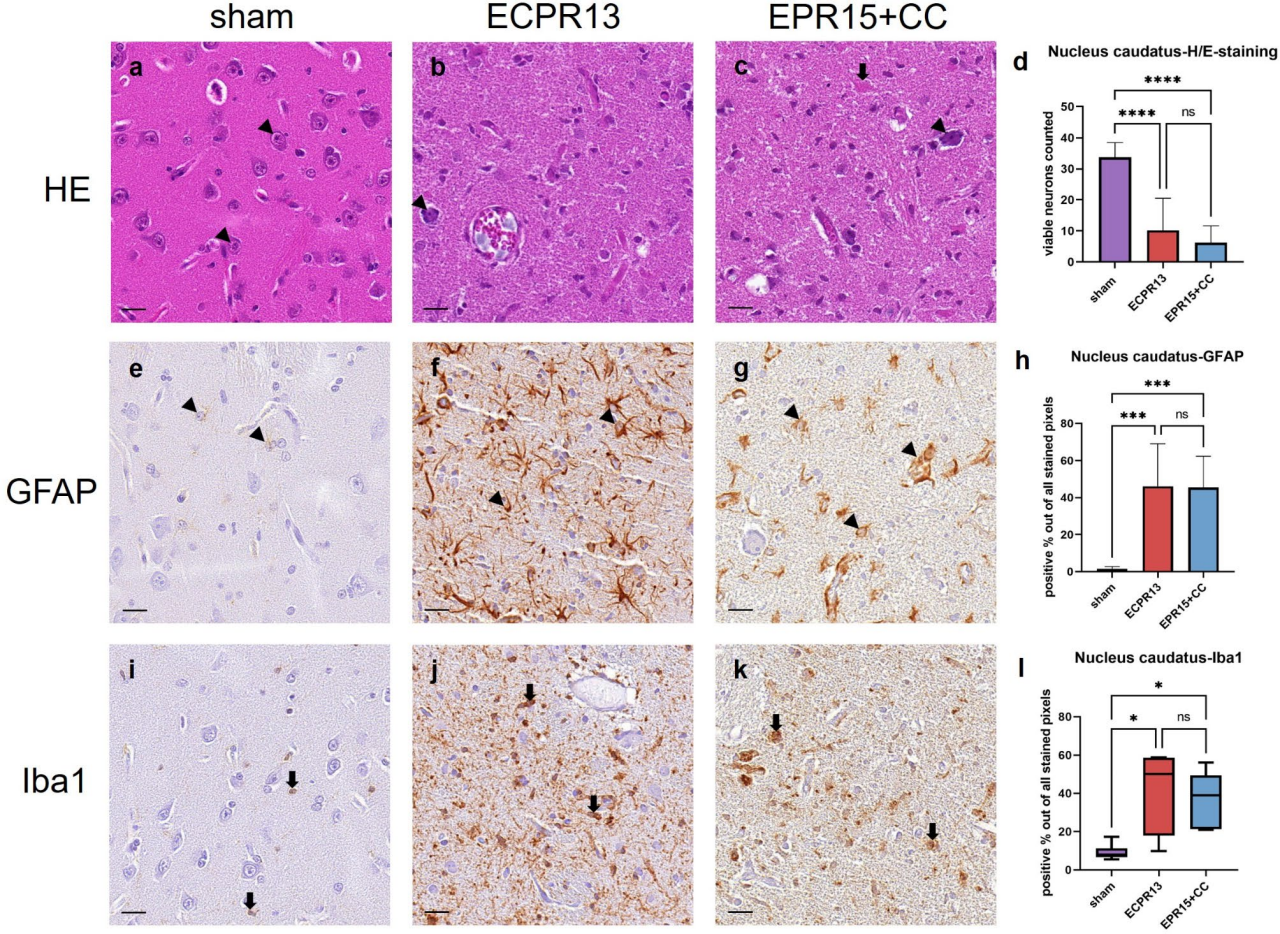
Representative images of the caudate nucleus in HE-staining, Iba1- and GFAP-immunohistochemistry. (**a**-**d**) HE-staining, caudate nucleus: (**a**) physiological appearance of the caudate nucleus in a sham animal with a high number of viable neurons (arrowheads); (**b**) one surviving neuron (arrowhead) in an ECPR13 pig; (**c**) an EPR15 + CC animal showing few viable (arrowhead) and necrotic (thick arrow) neurons; (**d**) the number of viable neurons was significantly higher in the caudate nucleus of sham animals compared to ischemic groups in HE-stained samples (****p < 0.0001). (**e-h**) GFAP-immunohistochemistry, caudate nucleus: (**e**) astrocytes showed mild GFAP immunoreactivity (arrowheads) in a sham animal; (**f**) strong labeling of astrocytes (arrowheads) in the caudate nucleus of an ECPR13 pig; (**g**) lesions and antibody signal (arrowheads) in the caudate nucleus of an EPR15 + CC pig; (**h**) GFAP signal was significantly higher in VFCA pigs than in sham pigs (***p < 0.001). (**i-l**) Iba1-immunohistochemistry, caudate nucleus: (**i**) physiologic Iba1 expression (thick arrows) in microglia cells of the caudate nucleus of a sham animal; (**j**,** k**) strong immunoreactivity (thick arrows) in both VFCA groups; (**l**) Iba1- expression was significantly higher in the caudate nucleus of VFCA pigs compared to sham animals (*p < 0.05). ns = no significant differences, bars = 20 μm.

In the caudate nucleus of sham animals medium sized neurons with round basophilic nuclei and one central nucleolus were visible. The caudate nuclei were lacking almost all viable neurons in VFCA pigs, which had been replaced by activated microglia and astrocytes (Fig. 4a-c). Numbers of viable neurons seemed to be increased with proximity to the lateral ventricle. Significantly higher numbers of viable neurons (ANOVA, *p* < 0.0001) could be found in sham pigs (*n* = 8) compared to VFCA animals (EPR15 + CC *n* = 6, ECPR13 *n* = 4) regardless of CA duration or resuscitation method (Fig. 4d). Astrocytes and microglia showed the same appearance as described in the cerebral cortex. Representative images of the immunohistochemistry in caudate nucleus are presented in Fig. 4e-g and i-k. Significantly more activation of microglia (Kruskal-Wallis, *p* = 0.0177 and *p* = 0.0127) and astrocytes (ANOVA, *p* = 0.0007 and *p* = 0.0003) was evident in VFCA pigs (EPR15 + CC *n* = 6, ECPR13 *n* = 4) compared to the sham group (*n* = 8) (Fig. 4h, l).

### Putamen

**Fig. 5:**
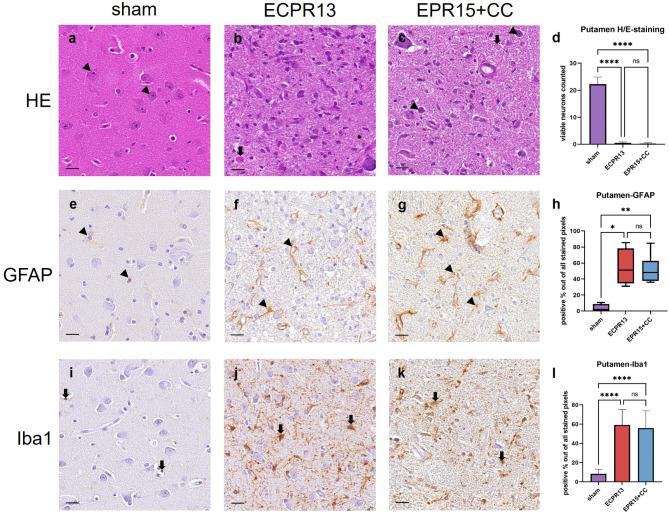
Representative images of the putamen in HE-staining, Iba1- and GFAP-immunohistochemistry.a-d HE-staining, putamen: (**a**) physiological appearance of the putamen in a sham animal with a high number of viable neurons (arrowheads); (**b**) eosinophil necrosis (thick arrow) in an ECPR13 pig; (**c**) an EPR15 + CC animal with eosinophil necrosis (thick arrow) and few viable neurons left (arrowheads); (**d**) the putamen showed significantly higher numbers of viable neurons in sham animals compared to ischemic groups in HE-stained samples (****p < 0.0001); (**e-h**) GFAP-immunohistochemistry, putamen: (**e**) physiologic GFAP expression in astrocytes (arrowheads) of a sham animal; (**f**),(** g**) strong GFAP expression in astrocytes (arrowheads) of an ECPR13 and an EPR15 + CC pig; **h** GFAP expression was significantly higher in VFCA pigs than in sham pigs (*p = 0.0110; **p = 0.0079). (**i-l**) Iba1-immunohistochemistry, putamen: (**i**) physiological appearance of the putamen in a sham animal with low Iba1 expression in microglia cells (thick arrows); (**j**,** k**) strong Iba1 immunoreactivity (thick arrows) in an ECPR13 and an EPR15 + CC animal; (**l**) Iba1 expression was significantly higher in the putamen of VFCA pigs compared to sham animals (****p < 0.0001). ns = no significant differences, bars = 20 μm.

In putamen shape and appearance of the neurons in sham animals were similar to the description given in the caudate nucleus. In VFCA animals, most neurons were also missing, and putamen was effaced by extraordinary high numbers of activated microglia and astrocytes, resulting in a marked increase in cellularity (Fig. 5a-c). Significantly lower numbers of viable neurons (ANOVA, *p* < 0.0001) were present in VFCA animals (EPR15 + CC *n* = 6, ECPR13 *n* = 4) compared to sham pigs (*n* = 8), regardless of CA duration or resuscitation method (Fig. 5d).

Astrocytes and microglia exhibited the same morphology as described in the cerebral cortex. Representative images of the immunohistochemical staining of the putamen are shown in Fig. 5e-g and i-k. The putamen of both arrest groups (EPR15 + CC *n* = 6, ECPR13 *n* = 4) showed significantly increased activation of astrocytes (Kruskal-Wallis, *p* = 0.0110 and *p* = 0.0079) and microglia (ANOVA, *p* < 0.0001) compared to the sham group (*n* = 8) (Fig. 5h, l).

### Hippocampus

**Fig. 6:**
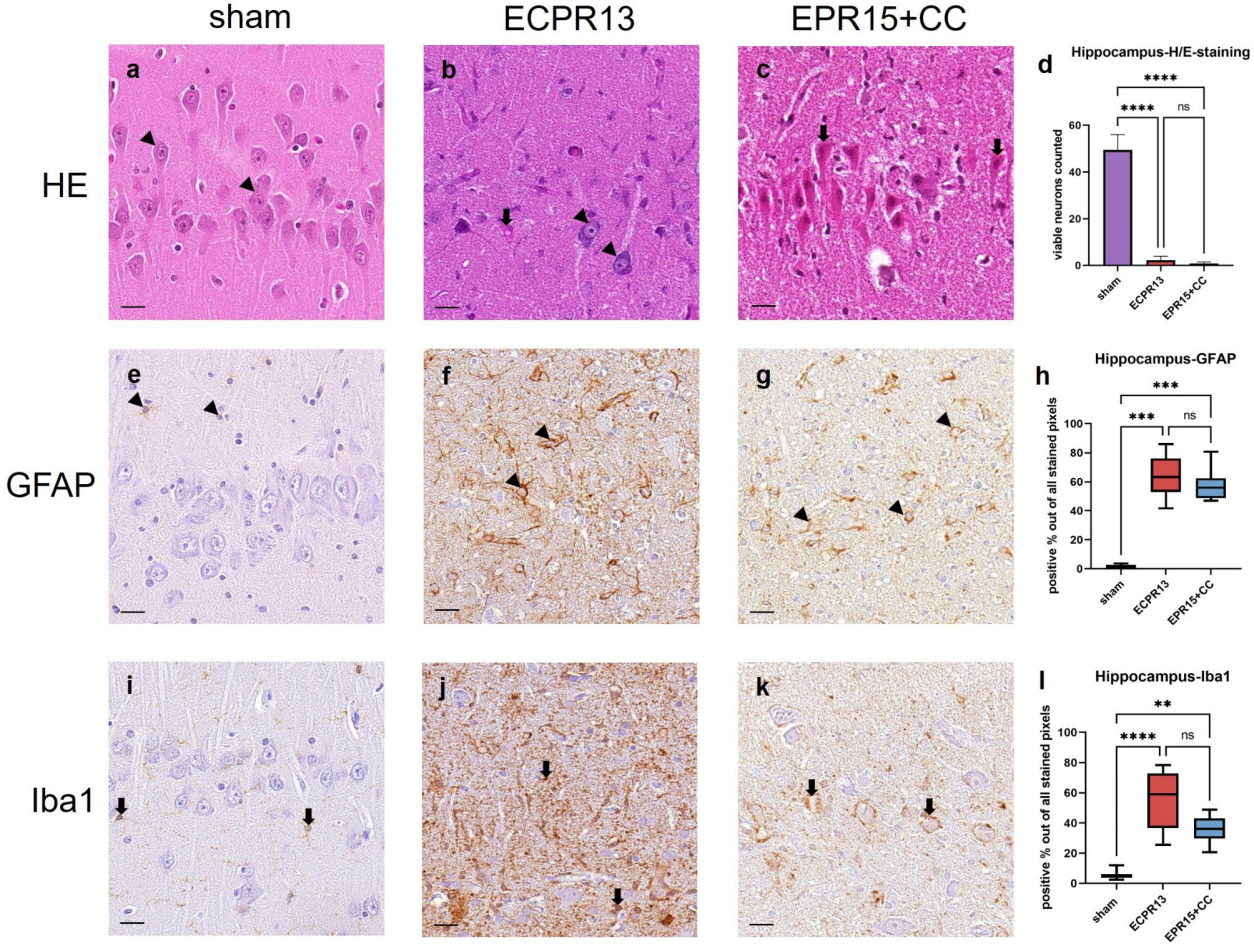
Representative images of the hippocampus in HE-staining, Iba1- and GFAP-immunohistochemistry. (**a**-**d**) HE-staining, hippocampus: (**a**) physiological appearance of a CA1 region in a sham animal with dense neuronal band (arrowheads); (**b**) necrosis (thick arrows) with few viable neurons (arrowheads) left in an ECPR13 pig; (**c**) necrosis of all pyramidal neurons (thick arrows) in an EPR15 + CC animal; (**d**) significantly more viable neurons could be found in sham animals compared to animals with cardiac arrest in HE stained samples (****p < 0.0001). (**e-h**) GFAP-immunohistochemistry, hippocampus: (**e**) CA1 region of the hippocampus in a sham animal with mild GFAP expression in astrocytes (arrowheads); (**f**,** g**) moderate GFAP expression (arrowheads) in the hippocampus of an ECPR13 and an EPR15 + CC pig; (**h**) significantly more GFAP expression was found in ischemic groups compared to sham animals of GFAP stained samples (***p < 0.0001). (**i-l**) Iba1-immunohistochemistry, hippocampus: (**i**) CA1 region in the hippocampus of a sham animal with mild immunoreactivity of microglia cells (thick arrows); (**j**) strong Iba1-signal (thick arrows) in the hippocampus of an ECPR13 animal; (**k**) increased Iba1 immunoreactivity (thick arrows) in the hippocampus of an EPR15 + CC pig; **l** Iba1 expression was significantly higher in VFCA groups compared to the sham group (****p < 0.0001; **p = 0.0027). ns = no significant differences, bars = 20 μm.

The pyramidal layer of the CA1 and CA2 regions of the hippocampus in sham pigs was characterized by a thick and dense band of neurons with large, lightly basophilic cell bodies and a big, round nucleus. One central, dark nucleolus could be seen in most of the nuclei (Fig. 6a). Hippocampi of VFCA animals were dominated by either marked neuronal necrosis, with neurons showing pink cytoplasm and shrunken dark nuclei, or missing neurons (Fig. 6b, c). Neuron numbers in the hippocampus were significantly reduced (ANOVA, *p* < 0.0001) in VFCA groups (EPR15 + CC *n* = 6, ECPR13 *n* = 4) compared to shams (*n* = 8) (Fig. 6d).

Morphology of astrocytes and microglia in sham and VFCA animals corresponded to the pattern described in caudate nucleus (Fig. 6e-g and i-k, respectively). Both VFCA groups (EPR15 + CC *n* = 6, ECPR13 *n* = 4) showed significantly increased areas of Iba1 (Kruskal-Wallis, *p* < 0.0001 and *p* = 0.0027) and GFAP (Kruskal-Wallis, *p* = 0.0001 and *p* = 0.0008) stained pixels compared to shams (*n* = 8) (Fig. 6h, l).

### Cerebellum

**Fig. 7:**
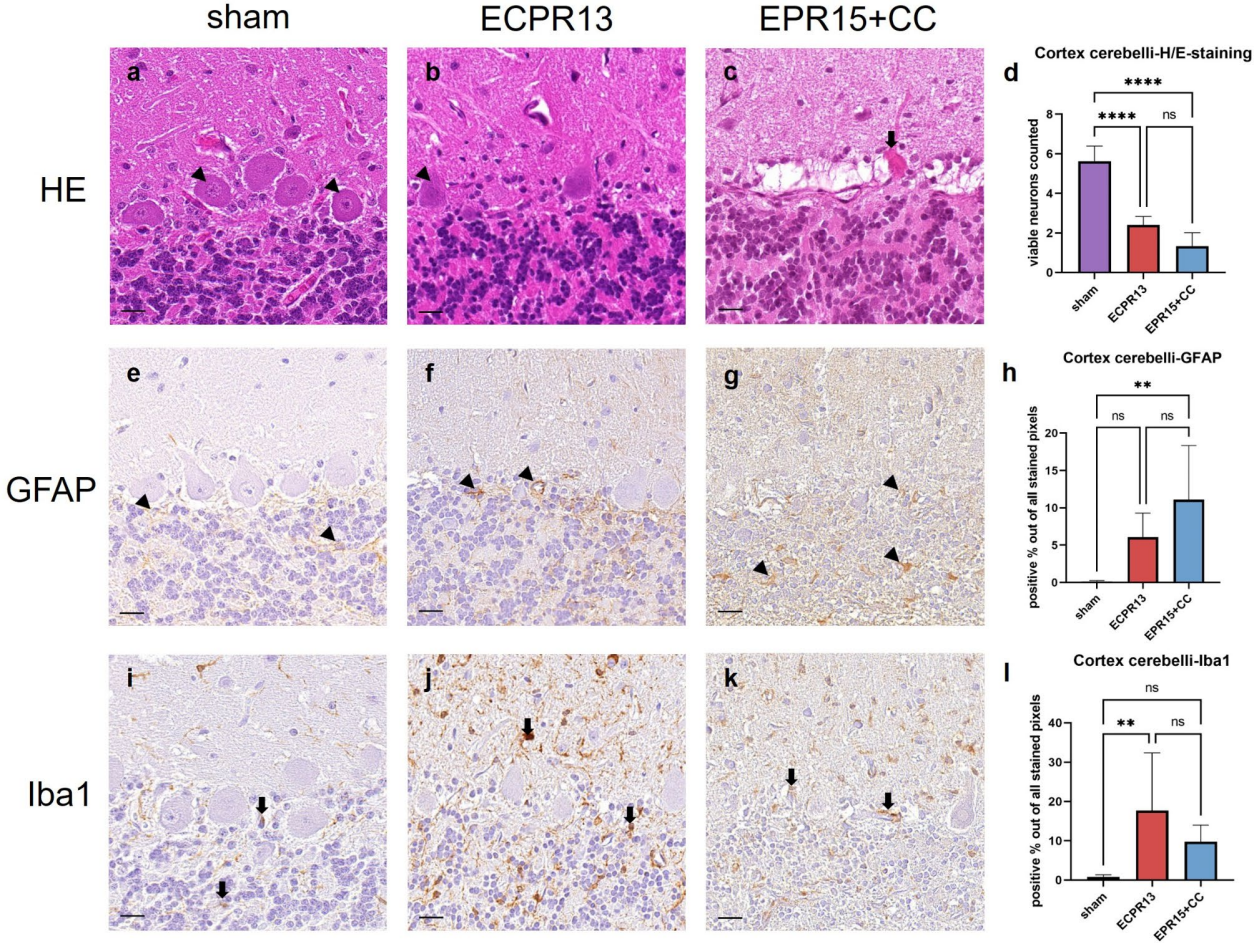
Representative images of the cerebellar cortex in HE-staining, Iba1- and GFAP-immunohistochemistry. a-d HE-staining, cerebellar cortex: (**a**) physiological appearance of the cerebellar cortex in a sham animal with multiple viable Purkinje cells (arrowheads); (**b**) few viable Purkinje cells (arrowhead) are present in an ECPR13 pig; **c** necrosis (thick arrow) in the Purkinje cell layer of an EPR15 + CC pig; (**d**) significantly more viable neurons are detected in sham animals compared to animals with VFCA in HE stained samples (****p < 0.0001). (**e-h**) GFAP-immunohistochemistry, cerebellar cortex: (**e**) physiologic GFAP immunoreactivity in astrocytes (arrowheads) of a sham animal; (**f**) mildly increased GFAP expression (arrowheads) in the cerebellar cortex of an ECPR13 animal; (**g**) moderate GFAP immunoreactivity (arrowheads) in the cerebellar cortex of an EPR15 + CC pig; (**h**) significantly more GFAP expression was present in the cerebellar cortex of EPR15 + CC pigs compared to sham animals (**p = 0.001). (**i-l**) Iba1-immunohistochemistry, cerebellar cortex: (**i**) Purkinje cell layer of the cerebellar cortex of a sham animal with scattered Iba1 positive microglia cells (thick arrows); (**j**) strong Iba1 immunoreactivity (thick arrows) in the cerebellar cortex of an ECPR13 animal; (**k**) moderate Iba 1 expression (thick arrows) in the cerebellar cortex of an EPR15 + CC pig; (**l**) Iba1 expression was significantly higher in the ECPR13 group than in sham animals (**p = 0.0036). ns = no significant differences, bars = 20 μm.

In the cerebellar cortex of sham pigs, the Purkinje cells were characterized by large, basophilic cell bodies with a high amount of cytoplasm and big, round nuclei with a central nucleolus. VFCA animals showed severe loss of Purkinje cells. Eosinophilic necrosis was also found, but to a lesser extent (Fig. 7a-c). Cell layers of the cortex were intact, regardless of the study group. The cortex revealed significantly lower numbers of viable Purkinje cells (ANOVA, *p* < 0.0001) in both VFCA groups (EPR15 + CC *n* = 6, ECPR13 *n* = 4) compared to sham animals (*n* = 8) (Fig. 7d).

The morphology of astrocytes and microglia cells of sham and VFCA pigs was similar to the description in cortical regions. Activated astrocytes in VFCA animals were found mainly within the granule cell layer and around vessels. Within the molecular layer the Iba1 positive microglia were increased in the VFCA groups. Representatives are shown in Fig. 7e-g and i-k. GFAP staining showed significantly increased astrocyte activation (ANOVA, *p* = 0.0010) in EPR15 + CC animals (*n* = 6) compared to shams (*n* = 8), while Iba1 staining revealed significant microglia activation (ANOVA, *p* = 0.0036) in ECPR13 animals (*n* = 4) compared to shams (*n* = 8) (Fig. 7h, l).

Since the cerebellar white matter contains no neurons physiologically, only immunohistochemistry was carried out. While activated astrocytes appeared as light brown spots with thin, long cell processes, and brown stained neuropil, activated microglia could be found as dark brown cells with thick, but short or no cell processes. Both stainings were less intense in EPR15 + CC pigs compared to ECPR13 pigs, but without statistical significance. In the cerebellar medulla, significantly more microglia activation (ANOVA, *p* = 0.0004 and *p* = 0.0184) could be detected in both VFCA groups (EPR15 + CC *n* = 6, ECPR13 *n* = 4) compared to sham pigs (*n* = 8). In contrast, only ECPR13 animals (*n* = 4) showed significantly increased activation of astrocytes (Kruskal-Wallis, *p* = 0.0039) compared to sham (*n* = 8), but not EPR15 + CC pigs (*n* = 6) (suppl. data, Fig. S4, e, j).

## Discussion

In the present study all animals subjected to VFCA showed a significant loss of viable neurons in all regions examined compared to sham animals. Although neuron numbers cannot be directly compared to HDS data, neuronal death leads to increased HDS values. Therefore, our results are in accordance with previous VFCA^17^ and ECPR studies^9^ in pigs, and EPR studies in dogs^5,6^. However, regarding the resuscitation method we did not find significant differences in neuron numbers between EPR15 + CC and ECPR13 animals. Therefore, we conclude that EPR does not directly protect neurons after VFCA.

EPR significantly reduced the inflammatory reaction in multiple cerebral cortex regions. Since the CPB duration of all groups was comparable, an increase of neurological inflammation due to prolonged CPB is negligible. Although the pigs with ECPR were subjected to only 13 min of VFCA, the temporal, parietal and occipital cortex showed significantly more activation of astrocytes than in EPR15 + CC pigs. Furthermore, significantly less activation of microglia cells was detected in the parietal cortex of EPR15 + CC animals compared to ECPR13 pigs. Since the overall performance score (OPC) and the neurologic deficit score (NDS) in the underlying studies of the present work showed differences between the EPR + CC and the ECPR groups^9,10^, we assume on the basis of the present results that variations in NDS and OPC might be consequences of the intensity of neuroinflammation. Comparable EPR studies in dogs^5,6,20^, pigs^7,9,21^, and mice^8^ primarily performed HDS analysis. Therefore, neuronal damage and inflammatory reaction cannot be distinguished in these semiquantitative approaches. Interestingly, similar studies in humans revealed either no significant clinical improvement of patients with intra-arrest cooling via intravenous flush or evaporative intra-nasal cooling^22,23^, or even negative impact of cold, intravenous infusions during resuscitation or after achieving ROSC^24,25^. However, these studies did not evaluate cerebral inflammatory reaction, but rather focused on clinical outcome.

In accordance with previous animal studies^13,14,26^, the hippocampus, followed by the putamen, could be identified as regions extremely sensitive to global ischemia with very low viable neuron numbers. Endisch et al.^27^ rated selective neuronal death in a semiquantitative manner in humans resuscitated from CA with CPR and found hypoxic ischemic encephalopathy to be most severe in hippocampus and cerebellum, followed by cerebral cortex. In accordance with their results, we also found neuron numbers to be lowest in the hippocampus. Cerebral cortex, caudate nucleus, and the cerebellar cortex also showed low neuronal survival, but quantitatively more viable neurons were evident than in hippocampus and putamen. However, the number of viable neurons did not differ significantly between EPR15 + CC pigs and ECPR13 animals in any region. This may be due to the late onset of the flush, long survival time and already manifest damage to neurons after 13 and 15 min of no-flow^17,28–30^. In comparison, studies in dogs found better HDS in the neocortex, hippocampus and cerebellum after exsanguination, two minutes of CA and 60 min EPR, but less effect in the striatum^5,6^. Missing energy storage of neurons and earlier dysfunction of astrocytes^31–33^, resulting in delayed cell death within days post resuscitation and reperfusion^29,30,34^, may further contribute to the observed loss of neurons. In contrast to the beneficial effect of EPR on the inflammatory reaction in the cerebral cortex, the hippocampus, the striatum and the cerebellar cortex revealed little or no evidence of reduced inflammation. Nevertheless, Drabek et al. (2009)^35^ found a significant decrease of microglial activation in the hippocampus and dentate gyrus of rats after prolonged exsanguination CA and deep flush hypothermia, without attenuating hippocampal neuronal death. However, different species, CA protocol, resuscitation protocol and survival time alter the comparability of this study. Moreover, microglial activation of EPR + CC pigs within our study was clearly decreased in the hippocampus compared to ECPR pigs but could not reach significance. This could possibly be achieved through a larger sample size.

The reduced glia cell activation in certain cortical regions could be caused by a downregulation of the metabolism due to EPR^36^, and partially substitution of the hypoxic blood with flush infusion. Therefore, less reactive oxygen species production post resuscitation and EPR could be possible, and thus less inflammatory reaction in the tissue.

A further explanation for variable patterns of immunological reactions of different brain regions to EPR could be disturbances of the microcirculation in certain brain areas post CA, which was first described by Ames et al. as “phenomenon of no-reflow” in 1968 ^37^. This phenomenon occurs at a total ischemia time starting from five minutes in cats^38^ to 7.5 min in rabbits^37^, and is manifested by a lack of perfusion in the capillaries of the brain, directly after ROSC^39,40^. Factors, such as decreased blood pressure and increased blood viscosity, edema formation, dehydration and postischemic acidosis are possible explanations for this phenomenon^40^. In cats, rabbits and monkeys, brainstem, basal ganglia, thalamus, and cerebellum are mainly affected, which is in accordance with our results. Although the cold flush in our studies contained heparin, vasopressin and epinephrine, the possibility remains that the regions highly vulnerable to no-reflow were not perfused sufficiently by the flush, and therefore the inflammatory reaction was increased. Furthermore, heparin does not contribute sufficiently to the improvement of the no-reflow phenomenon^37,41^. Years after the underlying studies of this work, Ristagno et al. (2007 and 2009)^42,43^ found, that the administration of epinephrine even had adverse effects on cerebral microvascular perfusion as well as on oxygen and carbon dioxide tension in pig brains after and during CPR of VFCA. Belohlavek et al. (2012)^44^ completely omitted vasopressors during an extracorporeal membrane oxygenation (ECMO) resuscitation approach in swine and achieved favorable outcome in cerebral and peripheral reoxygenation. However, the “no-flow” in this study was entirely substituted by an ECMO-generated “low-flow” time, and survival of the animals post ROSC was limited to 60 min. Nevertheless, it cannot be excluded, that epinephrine or vasopressin contribute to the different patterns of immunological reaction.

Interestingly, Iba1 staining showed less intense signal and the level of significance between groups was lower compared to GFAP staining. A possible explanation could be a time-dependent appearance and decrease of astrocytes and microglia cells, since microglia cells have been observed to sense damage, be activated and subside earlier than astrocytes after ischemic brain injury^45,46^. Moreover microglia are, beside endothelial cells^47^, essential for astrocyte activation and furthermore, for neuronal inflammation activation and inflammatory factor production^48,49^. Another possible explanation for the stronger and longer lasting reaction of activated astrocytes could be their dominant role in supporting delayed neurogenesis^50^ and glial scar formation^51^, involving astrogliosis and neuronal dependent functions^50,52^.

An additional, interesting finding of the study was the variability in astrocyte and microglia reaction within the cortical white matter. Astrocytes did not reveal any significant differences in activity except for the occipital medulla in VFCA animals. These conflicting results in GFAP staining in cerebral gray and white matter may suggest a variable reaction to ischemia between different groups of astrocytes, the protoplasmic in the gray matter and the fibrous in the white matter^53^. Also, in humans GFAP is expressed by non-reactive astrocytes in the white matter in a higher amount than in the gray matter^53,54^. In contrast, microglia in the cerebral white matter showed similar expression patterns as in the according gray matter areas, although differences did not always reach statistical significance.

Nevertheless, the performed study has several limitations. During the last years, ECPR resuscitation protocols changed and increased the survival rate in clinical trials^55,56^, achieving a favorable outcome with significantly longer CA times. Some even found no significant differences in survival between ECPR and conventional advanced cardiovascular life support (ACLS)^57,58^. Also, it has to be considered, that epinephrine was found to have adverse effects on cerebral perfusion in pigs during and after CPR^42,43^, and a vasopressor free approach of ECPR reached successful reoxygenation in pig brains^44^. Consequently, it must be mentioned, that the implementation of CPR from the previous, clinical studies does not correspond to the current guidelines^59,60^, particularly with regard to the compression rate and the drugs administered. With current resuscitation protocols, it is reasonable to hypothesize that a more favorable outcome in ECPR and even better in EPR + CC pigs is indeed a possibility. These considerations should be taken into account when estimating the translatability of the present study. Moreover, all animals included were healthy and had no previous diseases evident, in contrast to human CA patients, who often suffer from comorbidities. Because of the retrospective nature of the study, it was not possible to perform Western blot for verification of GFAP and Iba1 expression, due to missing material. Furthermore, only one brain side of each region could be examined, because material of the other half was not available. However, previous studies found that lesions after CA occur symmetrically^17^. Moreover, animal numbers per group were small, due to limited survival of the animals, and brains of animals that died before the endpoint were not collected, which may be reflected in statistical difficulties.

## Conclusions

We found that, even with a longer CA duration, animals resuscitated with EPR showed significantly less neuroinflammation in the cerebral cortex compared to ECPR pigs. This effect was not present in any other brain region. The method of resuscitation did not influence the extent of neuronal death, which was substantial in all VFCA animals. Moreover, possible effects of the EPR principle on other organ systems, such as the heart, might also influence the neurological outcome and survival of CA patients and should therefore be the subject of further investigations. Further research is also crucial to determine the underlying mechanisms of the protective EPR effect and understand the complex reactions and interactions in different brain regions after cardiac arrest and global ischemia.

## Electronic supplementary material

Below is the link to the electronic supplementary material.

Supplementary Material 1

## Data Availability

All data supporting the findings of this study are available from the corresponding author on reasonable request.
