# Supplementary material for: Cold aortic flush after ventricular fibrillation cardiac arrest reduces inflammatory reaction but not neuronal loss in the pig cerebral cortex
Source: Sci Rep. 2025 Apr 4;15:11659. doi: 10.1038/s41598-025-95611-9 (PMC11971268; doi:10.1038/s41598-025-95611-9)
Supplement: Supplementary file 1 — Supplementary Material 1 [file 41598_2025_95611_MOESM1_ESM.docx]

## **Cold aortic flush after ventricular fibrillation cardiac arrest reduces inflammatory reaction but not neuronal loss in the pig cerebral cortex**

Lisa Barones^1^, Wolfgang Weihs^2^, Alexandra Schratter^3^, Andreas Janata^2^, Petra Kodajova^1^, Helga Bergmeister^4^, Lukas Kenner^1, 5^, Michael Holzer^2^, Wilhelm Behringer^2^, Sandra Högler^1,*^

^1^Laboratory Animal Pathology, Department of Biological Sciences and Pathobiology, University of Veterinary Medicine Vienna, Vienna, Austria

^2^Department of Emergency Medicine, Medical University of Vienna, Vienna, Austria

^3^Department of Cardiology, Klinik Floridsdorf, Vienna, Austria

^4^ Center for Biomedical Research and Translational Surgery and Ludwig Boltzmann Institute for Cardiovascular Research, Medical University Vienna, Vienna, Austria

^5^ Clinical Institute of Pathology, Department for Experimental and Laboratory Animal Pathology, Medical University of Vienna, Vienna, Austria

**Supplementary** **Data**

**Table 1:** Antibodies used for immunohistochemistry.

| Antibody | Catalogue number | Dilution | Animal | Polyclonality | Company |
| --- | --- | --- | --- | --- | --- |
| GFAP | #Z0334 | 1:4000 | rabbit | polyclonal | Agilent Dako, Santa Clara, USA |
| Iba1 | #019-19741 | 1:1000 | rabbit | polyclonal | FUJIFILM Wako Chemicals U.S.A. Corporation, Richmond, USA |


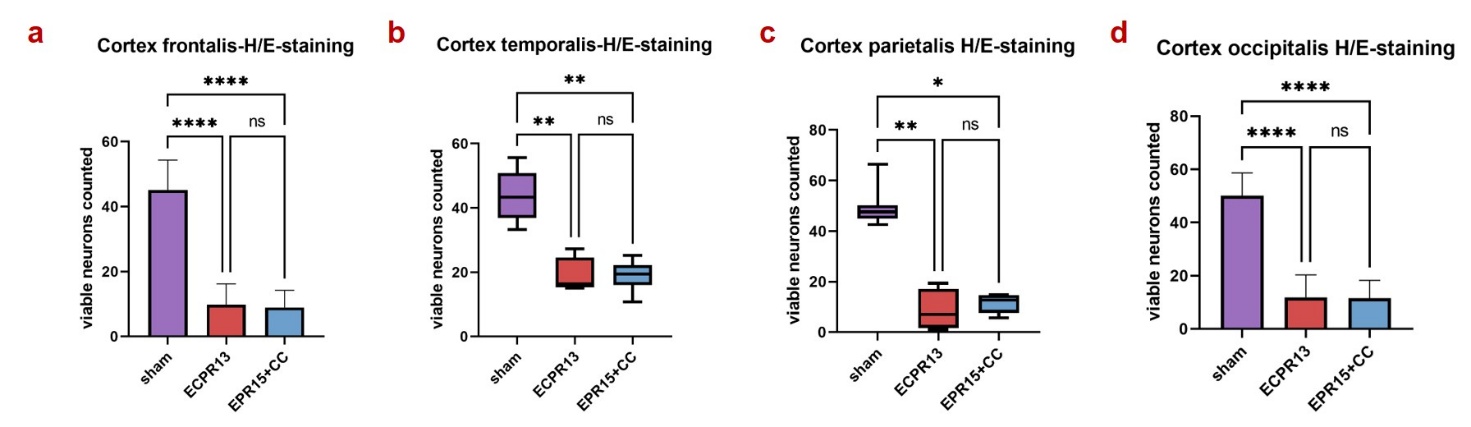
**Fig. 1. Quantitative evaluation of cortical regions in H/E-staining.**

Quantitative evaluation through neuron counting of frontal (A), temporal (B), parietal (C) and occipital (D) cortex of sham animals (n=8), ECPR13 animals (n=4), and EPR15+CC animals (n=6). Correlation of the strength of significance with the number of asterisks (****, p< 0.0001; ***, 0.0001≤ p< 0.001; **, 0.001≤ p< 0.01, *, 0.01≤ p< 0.05) with significantly higher number of viable neurons in sham animals compared to ischemic animals in all groups examined (A-D).


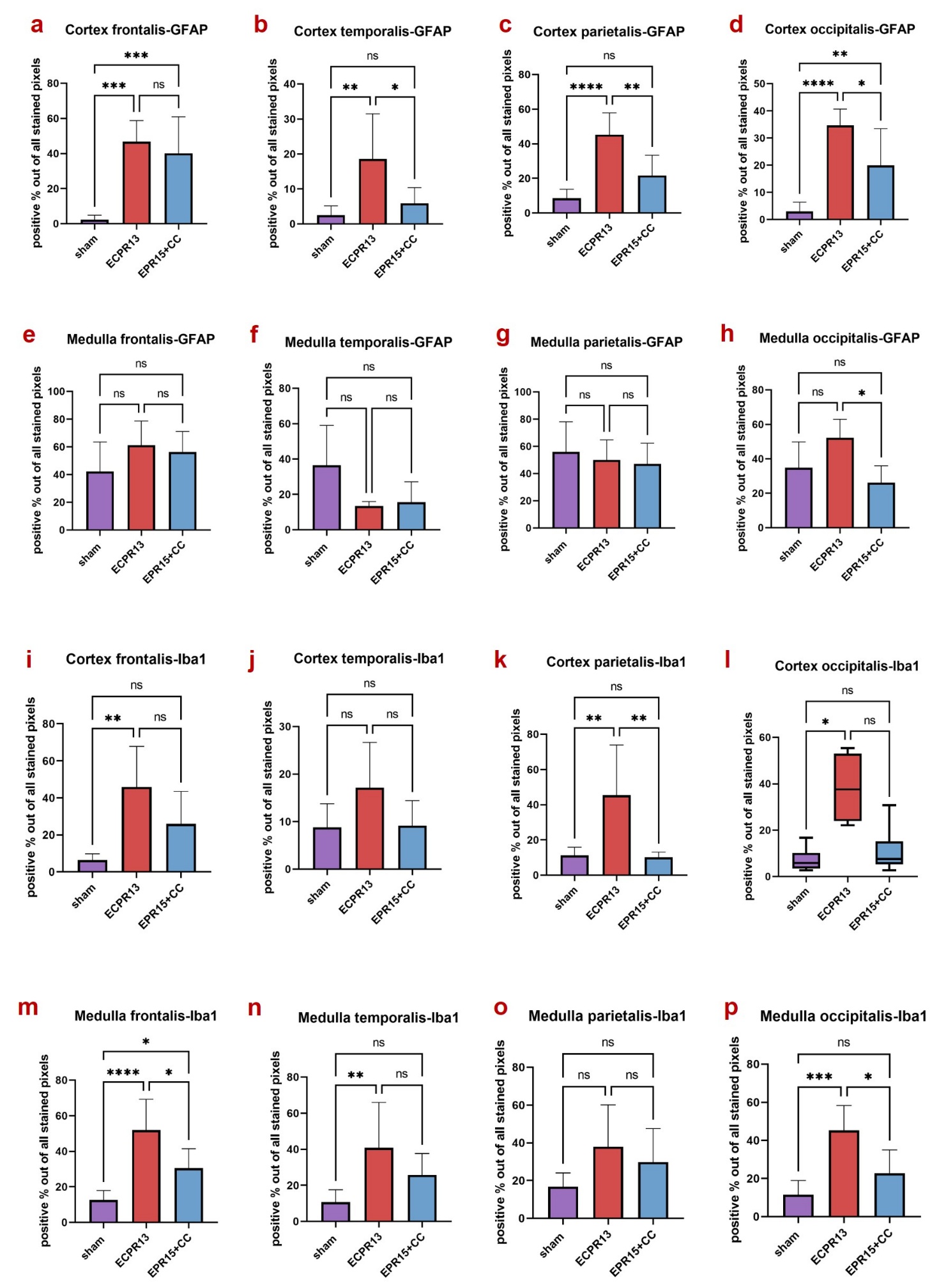


**Fig. 2** **Quantitative evaluation of GFAP- and Iba1-immunohistochemistry of cortical regions with QuPath.**

Quantitative evaluation of frontal (A, E, I, M), temporal (B, F, J, N), parietal (C, G, K, O) and occipital (D, H, L, P) cortex of sham animals (n=8), ECPR13 animals (n=4), and EPR15+CC animals (n=6) via the pathological image analysis program QuPath. Correlation of the strength of significance with the number of asterisks (****, p< 0.0001; ***, 0.0001≤ p< 0.001; **, 0.001≤ p< 0.01, *, 0.01≤ p< 0.05). Significantly lower antibody reaction in cortices of sham animals compared to ischemic groups in GFAP-samples of cortices, except for the parietal and temporal cortices of EPR15+CC pigs (A-D). Significantly less positive GFAP-reaction in EPR15+CC pigs of temporal, parietal and occipital cortices than in 13 EPR pigs, but not in frontal cortices (A). No significant differences in medullae of any group in GFAP-immunohistochemistry, except for less antibody reaction in EPR15+CC pigs compared to ECPR13 animals in the occipital medulla (E-H). Significantly lower positive Iba1 reaction in frontal, parietal and occipital cortex of sham animals compared to ECPR13 pigs (I, K, L). Additional significantly more positive Iba1 reaction in parietal cortices of ECPR13 pigs than in EPR15+CC pigs (K). No significant differences (ns) among the groups in temporal cortices (J). Frontal medulla in Iba1 samples with significant differences among all groups examined (M). Significantly lower antibody reaction in medulla temporalis and occipitalis of sham pigs compared to ECPR13 pigs and additionally more positive pixels in occipital medullae of ECPR13 pigs than EPR15+CC pigs (N, P). No significant differences among groups in parietal medullae (O).


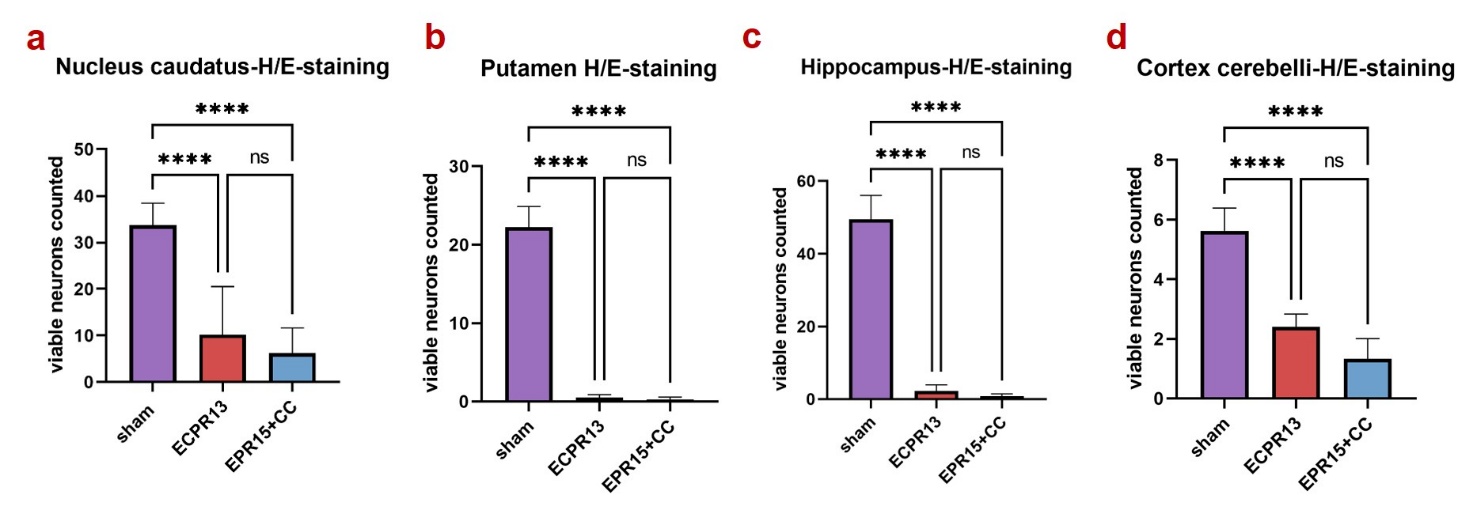


**Fig. 3. Quantitative evaluation of striatum, hippocampus, and cerebellum in H/E staining.**

Quantitative evaluation through neuron counting of striatum (A, B), hippocampus (C) and cerebellum (D) of sham animals (n=8), ECPR13 animals (n=4), and EPR15+CC animals (n=6). Correlation of the strength of significance with the number of asterisks (****, p< 0.0001; ***, 0.0001≤ p< 0.001; **, 0.001≤ p< 0.01, *, 0.01≤ p< 0.05). Significantly more viable neurons in sham animals compared to VFCA pigs in every brain region examined (A-D).


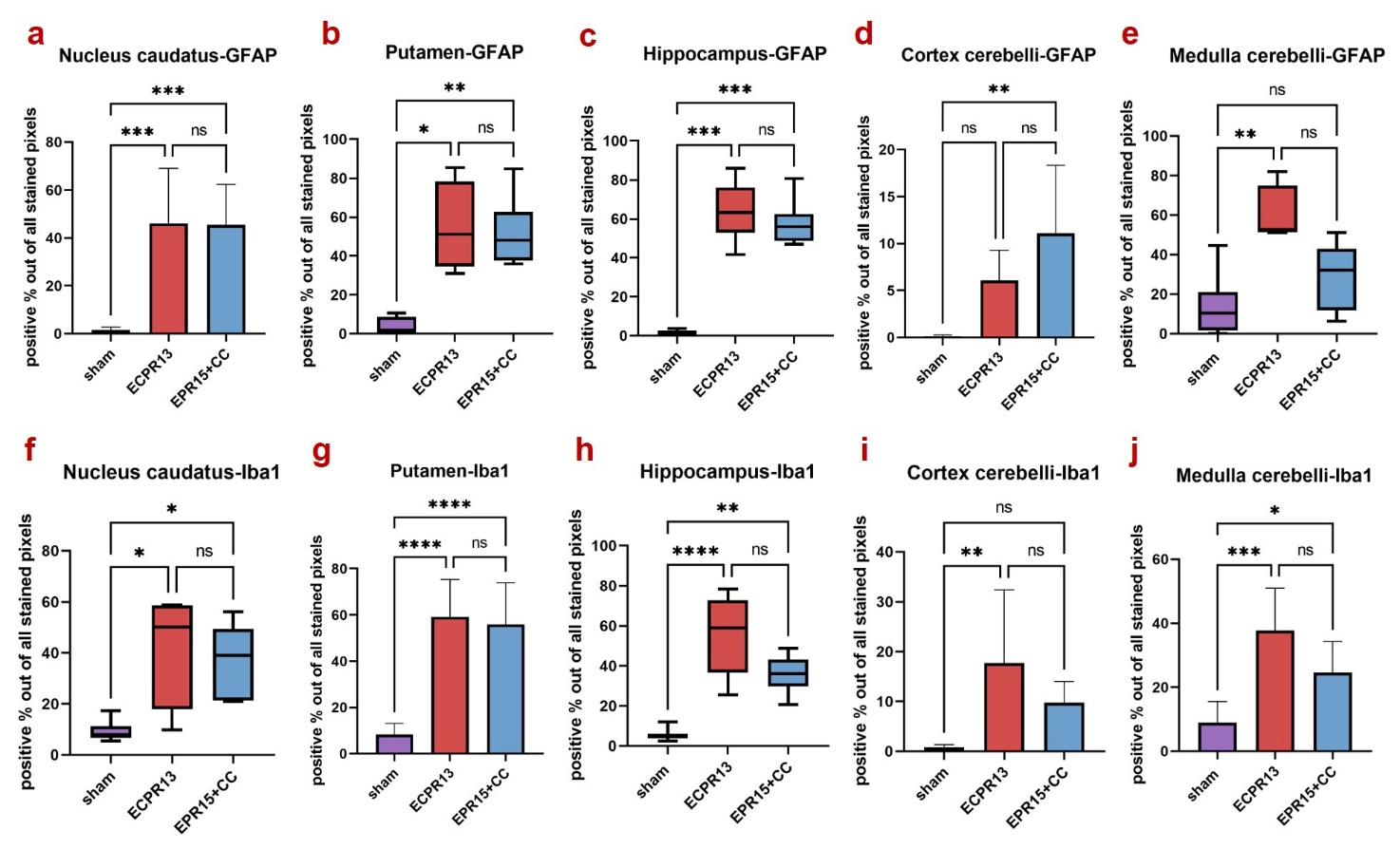


**Fig. 4. Quantitative evaluation of GFAP- and Iba1-immunohistochemistry of striatum, hippocampus, and cerebellum with QuPath.**

Quantitative evaluation of striatum (A, B, F, J), hippocampus (C, K) and cerebellum (D, E, I, J) of sham animals (n=8), ECPR13 animals (n=4), and EPR15+CC animals (n=6) via the pathological image analysis program QuPath. Correlation of the strength of significance with the number of asterisks (****, p< 0.0001; ***, 0.0001≤ p< 0.001; **, 0.001≤ p< 0.01, *, 0.01≤ p< 0.05). Significantly lower Iba1- and GFAP-reaction in striatum of sham animals (n=8) compared to ischemic animals (A, B, F, G), except for Iba1 reaction of the caudate nucleus in ECPR13 pig (F). Significantly lower antibody reaction in hippocampus of sham animals compared to ischemic groups in Iba1 and GFAP stained samples (C, H). Significantly less activation of astrocytes in sham animals than in the cerebellar cortex of EPR15+CC pigs (D), and less activation of microglia cells in sham pigs compared to ECPR13 pigs (I). Significantly lower GFAP-reaction in sham pigs compared to ECPR13 pigs in the cerebellar medulla (E). Also, significantly lower positive Iba1-reaction in sham animals compared to cerebellar medullae of VFCA animals (J).


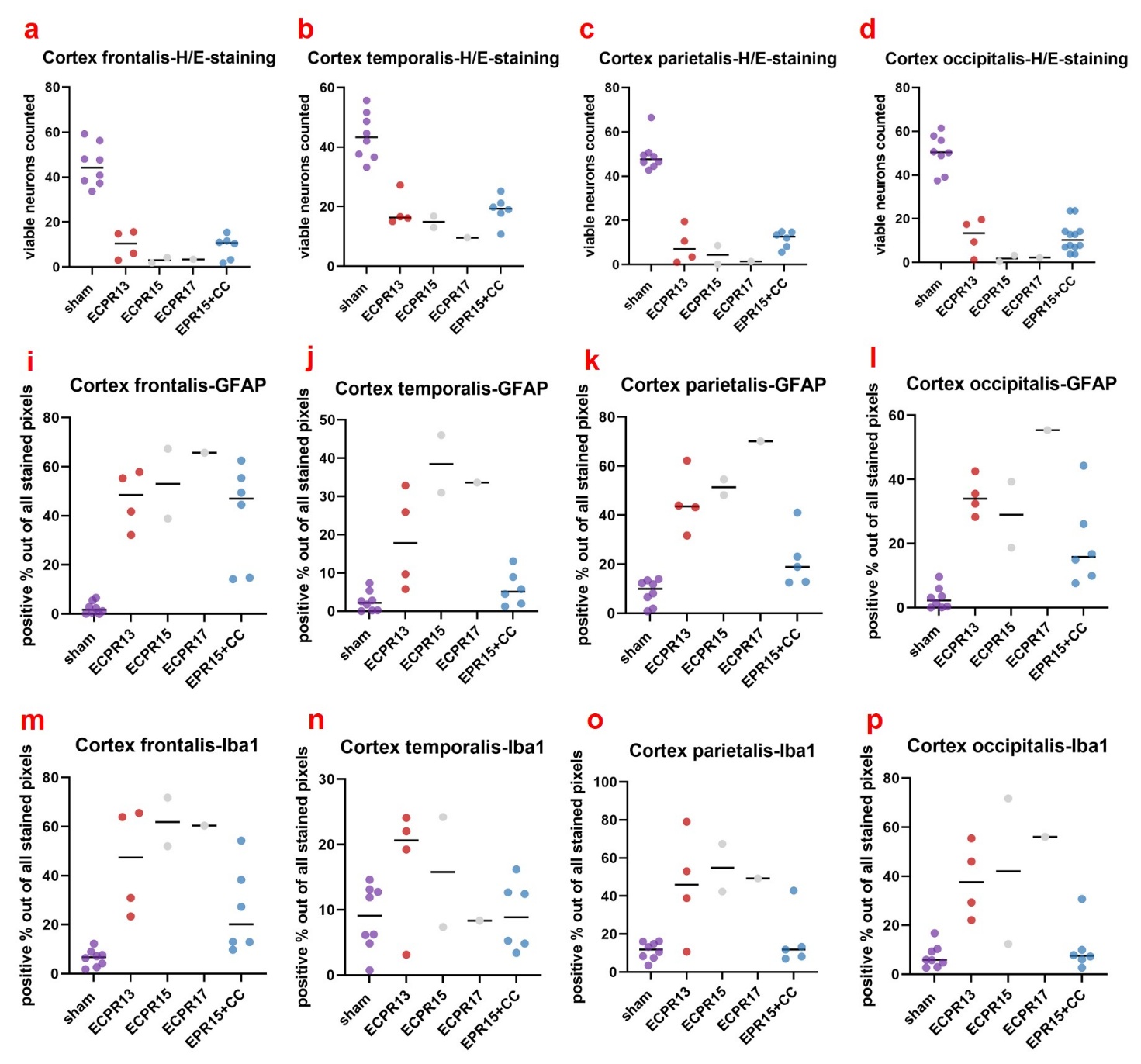
**Fig. 5. Representation of the trends in cortical regions of 15 and 17 ECPR animals in H/E-staining, GFAP-and Iba1-immunohistochemisty.**

Tending to equal viable neuron numbers in all cortical areas of all examined groups of HE stained slides (A-D). Tending to highest positive GFAP-reaction in cortices of 17 ECPR pigs, except for the frontal cortex (I-L). Tending to highest Iba1-reaction in cortices of 15 ECPR animals (M-P).
